# Gas signatures from *Escherichia coli* and *Escherichia coli*-inoculated human whole blood

**DOI:** 10.1186/2001-1326-2-13

**Published:** 2013-07-10

**Authors:** Brandon J Umber, Hye-Won Shin, Simone Meinardi, Szu-Yun Leu, Frank Zaldivar, Dan M Cooper, Donald R Blake

**Affiliations:** 1 Department of Chemistry, University of California, Irvine, CA 92697, USA; 2 Department of Pediatrics, University of California, Irvine, CA 92697, USA; 3 Institute for Clinical and Translational Sciences, University of California, Irvine, CA 92697, USA

**Keywords:** Gas biomarker, Volatile organic compounds (VOCs), *Escherichia coli*, Cell culture, Human whole blood culture, Dimethyl sulfide (DMS), Carbon disulfide (CS_2_), Ethanol, Acetaldehyde, Methyl butanoate

## Abstract

**Background:**

The gaseous headspace above naïve *Escherichia Coli* (*E. coli*) cultures and whole human blood inoculated with *E. coli* were collected and analyzed for the presence of trace gases that may have the potential to be used as novel, non-invasive markers of infectious disease.

**Methods:**

The naïve *E. coli* culture, LB broth, and human whole blood or *E. coli* inoculated whole blood were incubated in hermetically sealable glass bioreactors at 37°C for 24 hrs. LB broth and whole human blood were used as controls for background volatile organic compounds (VOCs). The headspace gases were collected after incubation and analyzed using a gas chromatographic system with multiple column/detector combinations.

**Results:**

Six VOCs were observed to be produced by *E. coli*-infected whole blood while there existed nearly zero to relatively negligible amounts of these gases in the whole blood alone, LB broth, or *E. coli-*inoculated LB broth. These VOCs included dimethyl sulfide (DMS), carbon disulfide (CS_2_), ethanol, acetaldehyde, methyl butanoate, and an unidentified gas S. In contrast, there were several VOCs significantly elevated in the headspace above the *E. coli* in LB broth, but not present in the *E. coli*/blood mixture. These VOCs included dimethyl disulfide (DMDS), dimethyl trisulfide (DMTS), methyl propanoate, 1-propanol, methylcyclohexane, and unidentified gases R2 and Q.

**Conclusions:**

This study demonstrates 1) that cultivated *E. coli* in LB broth produce distinct gas profiles, 2) for the first time, the ability to modify *E. coli*-specific gas profiles by the addition of whole human blood, and 3) that *E. coli*-human whole blood interactions present different gas emission profiles that have the potential to be used as non-invasive volatile biomarkers of *E. coli* infection.

## Background

Over the past decade, the interest in using exhaled gases as non-invasive biomarkers in clinical diagnostics and therapeutic monitoring has steadily increased [1]. Considerable efforts have been made to determine clinically applicable breath biomarkers for such conditions as asthma, diabetes, and various cancers [2-7]. Evidence also exists that cultivated microorganisms can generate species or genus-specific profiles of VOCs [8-13]. Additionally, unique gas signatures have been observed in the exhaled breath of patients with specific infections, including bronchopneumonia [14] and *Helicobacter pylori*[15].

Identifying and quantifying gases in the exhaled human breath provide a non-invasive means of obtaining information about host responses to infection. However, with the exception of nitric oxide, the cellular sources or mechanisms responsible for the observed gases have not been fully investigated. It is conceivable that the host immune response may yield gases unique to one pathogen or another. *Escherichia Coli* (*E. coli*) was used as a model infectious organism to 1) determine the unique gas profile of bacteria in a “naïve” state and 2) investigate the VOCs that result from the bacteria and whole blood interactions. VOC profiles found in the interaction of bacteria and immune cells containing whole blood may then provide a beginning interpretation of the exhaled breath gases from infected subjects.

A hermetically sealable bioreactor was used to grow the cultures and collect the gaseous headspace. The headspace was then analyzed for trace gases produced and released by the microorganisms growing in culture using a multi-column and multi-detector analytical gas chromatography (GC) methodology [16].

This approach will soon be extended to additional infectious agents and it is anticipated that the technology will eventually demonstrate the capability to discriminate between multiple infectious pathogens by means of their unique VOC profiles.

## Methods

*Blood Sampling*. The blood was drawn using EDTA-treated vacutainers from adult male subjects through the Institute for Clinical Translational Sciences (ICTS) general blood donor program. Subjects having a history of any chronic medical conditions or use of any medications were excluded from the study. The Institutional Review Board at the University of California, Irvine, approved the study.

### E. Coli culture and headspace Gas collection

*E. coli* was purchased from Invitrogen (One Shot TOP10 Chemically Competent, Invitrogen, NY). The genotype of this *E. coli* is similar to DH10B strain. The complete genome sequence has been reported elsewhere (http://www.ncbi.nlm.nih.gov/pubmed/18245285). Gibco LB broth from Invitrogen was used for the study. The vials of *E. coli-*inoculated whole blood (1 × 10^6^*E. coli*/10 mL whole blood) and naïve *E. coli* inoculated LB broth (1 × 10^6^*E. Coli*/10 mL LB) were placed inside bioreactors specifically designed to collect the gaseous headspace above aqueous cultures [16]. For comparison, vials of LB broth only (10 mL) or vials of whole blood only (10 mL) were also placed inside bioreactors. To minimize ambient gaseous contaminants, each bioreactor was flushed with whole air containing low levels of VOCs and 5% CO_2_ for 10 minutes at 125 mL per minute. The bioreactors were then placed in an incubator at 37°C for 24 hrs. The headspace gases were collected after incubation and analyzed using a gas chromatographic system with multiple column/detector combinations [16-18]. Following sample collection, the bioreactor was disassembled and the cells were immediately collected and counted.

### GC system

The analyses of the headspace gases were performed on a system previously developed to measure trace atmospheric gases [17]. Briefly, a 233 cm^3^ (at STP) sample was cryogenically pre-concentrated and injected into a multi-column/detector GC system. The system consists of three Hewlett-Packard 6890 GC units (Wilmington, DE) using a combination of columns and detectors capable of separating and quantifying hundreds of gases, including, but not limited to, non-methane hydrocarbons (NMHCs), alkyl nitrates, and halocarbons in the ppbv to pptv range (10^-9^ -10^-12^). The detectors include flame ionization detectors (FIDs), electron capture detectors (ECDs), and a mass selective detector (MSD). Preliminary identifications of the unknown compounds were made using GC-MSD and verification was obtained by injecting the diluted headspace of pure compounds (Sigma-Aldrich, St. Louis, MO) to ensure that both the elution time and the mass spectrum matched that of the unknown.

### Data analysis

Four study conditions, Broth only, Broth + *E. coli*, Blood only, and Blood + *E. coli* were evaluated in each of the 10 experimental days. The same blood sample and *E. coli* sample were used in the same day. A total of 117 gases were examined for each sample. Thirty-eight gases were excluded; of these, 31 gases were below the limit of detection in over 80% of the samples and seven gases had concentrations less than 100 pptv in all samples. For any gas that could not be detected in a sample, the level was set to zero Wilcoxon signed-rank test, a non-parametric method for paired data, was used to compare the difference between Broth only and Broth + *E. coli*, between Blood only and Blood + *E. coli*, between Broth + *E. coli* and Blood + *E. coli*, and between Broth only and Blood only. The method was chosen because the sample size was small, the distributions of most gases were highly skewed, and the experiments performed on the same day were considered more correlated than those performed on different days. Multiple-comparison adjustment was further applied using Benjamini and Hochberg’s false discover rate (FDR) method [19]. A comparison was considered significant if FDR < 0.01. All analyses were performed using SAS 9 (Cary, NC) and all data were presented with median and range (minimum, maximum).

## Results

**Figure 1:**
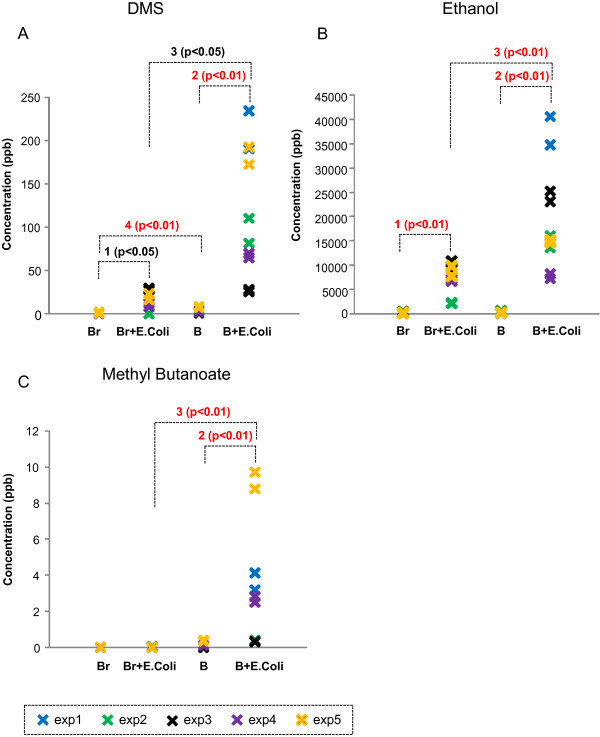
**Representative patterns of VOCs in Category 1. (A)** DMS, **(B)** Ethanol, and **(C)** Methyl Butanoate emission from 4 different conditions: broth only (Br), *E. coli* in LB broth (Br + *E. coli*), whole blood only **(B)**, and *E. coli*-inoculated whole blood (B + *E. coli*). 1. significantly different between Broth only and Broth + *E. coli*; 2: significantly different between Blood only and Blood + *E. coli*; 3: significantly different between Broth + *E. coli* and Blood + *E. coli*; 4: significantly different between Broth only and blood only.

**Figure 2:**
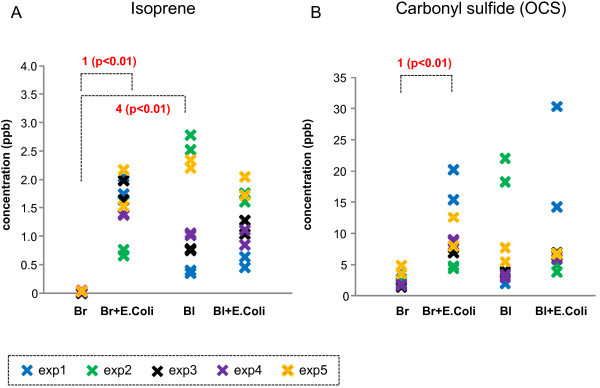
**Representative patterns of VOCs in Category 2. (A)** Isoprene and **(B)** carbonyl sulfide (OCS) emission from 4 different conditions: only (Br), *E. coli* in LB broth (Br + *E. coli*), whole blood only **(B)**, and *E. coli*-inoculated whole blood (B + *E. coli*). 1: significantly different between Broth only and Broth + *E. coli* i; 2: significantly different between Blood only and Blood + *E. coli* i; 3: significantly different between Broth + *E. coli* and Blood + *E. coli*; 4: significantly different between Broth only and blood only.

**Figure 3:**
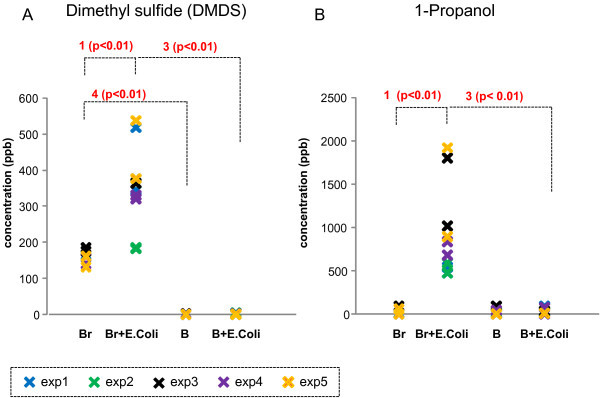
**Representative patterns of VOCs in Category 3. (A)** Dimethyl disulfide (DMDS) and **(B)** 1-propanol emission from 4 different conditions such as broth only (Br), *E. coli* in LB broth (Br + *E. coli*), whole blood only **(B)**, and *E. coli*-inoculated whole blood (B + *E. coli*). 1: significantly different between Broth only and Broth + *E. coli*; 2: significantly different between Blood only and Blood + *E. coli*; 3: significantly different between Broth + *E. coli* and Blood + *E. coli*; 4: significantly different between Broth only and blood only.

**Figure 4:**
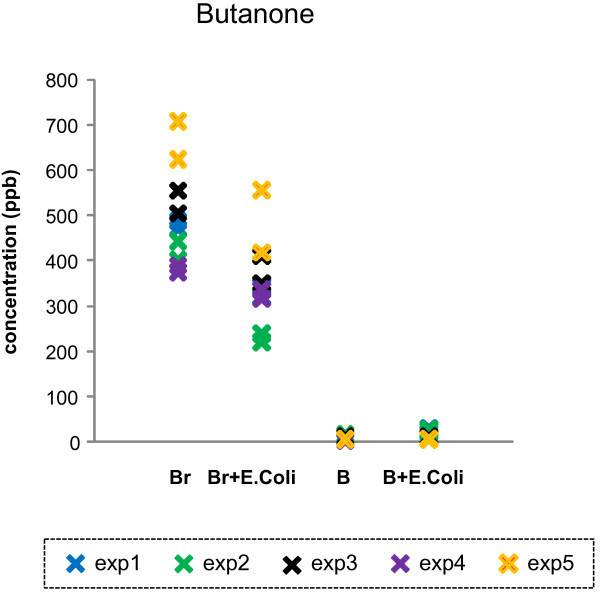
**Butanone emission from 4 different conditions such as broth only (Br), *E. coli *in LB broth (Br + *E. coli*), whole blood only (B), and *E. coli *-inoculated whole blood (B + *E. coli*).** Butanone is an exemplary gas that originates from LB broth.

**Figure 5:**
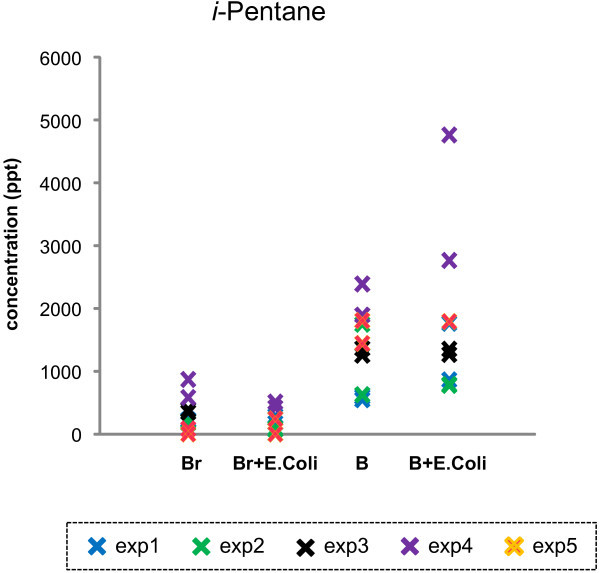
***i*-Pentane emission from 4 different conditions such as broth only (Br), *E. coli *in LB broth (Br + *E. coli*), whole blood only (B), and *E. coli*-inoculated whole blood (B + *E. coli*).***i*-Pentane is an exemplary gas that originates from human whole blood.

After statistical analysis, 40 gases were allocated into five categories based on the pattern of gas release: category 1: VOCs elevated from *E. coli-*inoculated human whole blood, (see Table 1 and Figure 1); category 2: VOCs elevated from *E. coli* in LB broth (see Table 1 and Figure 2); category 3: VOCs elevated from naïve *E. coli* in broth, but decreased by *E*. *coli-*inoculated whole blood (see Table 1, and Figure 3); category 4: VOCs mainly from pure LB broth only (see Table 2 and Figure 4); category 5: VOCs mainly from human whole blood only (see Table 3 and Figure 5).

**Table 1:** VOCs of interest

**Gas**	**Stat. diff.**	**Broth**		**Blood**	
			**+ *E. coli***		**+ *E. coli***
**Category 1: VOCs elevated in *E. coli*-infected blood**					
Diemthyl sulfide [pptv]	2,4	970 (310,2310)	16010 (180,29500)	3770 (1250,8820)	95920 (25740,234400)
Carbon disulfide [pptv]	2,3,4	85 (55,470)	295 (80,790)	4390 (2450,10040)	8940 (2620,16550)
Acetaldehyde [ppbv]		736 (558,1089)	645 (44,1359)	45 (22,145)	1158 (208,3493)
Ethanol [ppbv]	1,2,3	202 (28,564)	8388 (2037,10867)	218 (5,604)	15486 (7199,40441)
Unk S^§^	2,3	31 (0,316)	74 (12,267)	675 (0,3311)	15001 (2208,37426)
Methyl Butanoate [pptv]		0 (0,25)	0 (0,40)	60 (0,405)	2675 (330,9720)
**Category 2: VOCs elevated in naïve *E. coli *in LB broth**					
Isoprene [pptv]	1,4	0 (0,50)	1590 (665,2175)	1040 (350,2785)	1200 (455,2050)
Carbonyl sulfide (OCS) [pptv]	1	2240 (1440,4850)	8360 (4360,20180)	3850 (1970,22040)	6540 (3810,30340)
Unk R1^§^		7474 (1568,42622)	116738 (15070,1005677)	3758 (105,20243)	2435 (785,10277)
**Category 3: VOCs elevated in naïve *E. coli *but substantially lower from co-culture of *E. coli *and whole blood**					
Dimethyl disulfide [ppbv]	1,3,4	164 (133,185)	354 (182,537)	0.7 (0.3,2)	2 (0.6,4)
Methyl Propanoate [pptv]		3300 (2100,4300)	14500 (5660,21000)	400 (0,1400)	600 (0,1100)
Dimethyl trisulfide [pptv]	1,3	2900 (1900,9700)	160400 (22700,253300)	800 (10,6900)	400 (150,5400)
Unk_Q^§^		2052 (0,13997)	57320 (6317,467654)	620 (0,6232)	289 (0,3787)
1-Propanol [pptv]		175 (0,90200)	757300 (472900,1920800)	700 (0,93600)	33400 (4100,90200)
Methylcylohexane [pptv]		220 (60,990)	1600 (850,3200)	130 (0,1720)	170 (80,980)
Unk R2^§^		416 (0,5269)	15852 (2228,152789)	767 (0,9829)	674 (0,8522)

1: significantly different between Broth only and Broth + *E. coli*; 2: significantly different between Blood only and Blood + *E. coli*; 3: significantly different between Broth + *E. coli* i and Blood + *E. coli*; 4: significantly different between Broth only and blood only.

Data were presented with median and range (minimum, maximum).

^§^unknown peaks were presented as area under the curve.

**Table 2:** Gases mainly from pure broth

**Gas**	**Category 4 Gases mainly from LB broth (significantly higher in broth only compared to whole blood only)**			
	**Broth**		**Blood**	
		***+ E. coli***		**+ *E. coli***
Ethyl nitrate [pptv]	429 (371, 448)	233 (32,284)	28 (17,38)	28 (21,35)
Methyl nitrate [pptv]	705 (670, 825)	446 (38,534)	29 (27,35)	29 (26,36)
2-Propyl nitrate [pptv]	162 (147,177)	103 (30,125)	20 (14,29)	20 (18,33)
2-Methylpropanal [pptv]	90900 (56900,203300)	4300 (700,8900)	2010 (100,600)	600 (200,1300)
3-Methylhexane [pptv]	2000 (1120, 3290)	1020 (660,1660)	510 (400,1850)	530 (220,1410)
2,2,4-Trimethylpentane [pptv]	9140 (8030, 9940)	6100 (1660,7140)	700 (360,1220)	810 (620,1160)
*n*-Decane [pptv]	100 (70,140)	50 (30,50)	60 (30,70)	60 (30,80)
Benzene [pptv]	93640 (50700,114580)	25910 (300,72270)	1100 (0,2070)	1030 (440,1550)
Toluene [pptv]	98930 (54460,129080)	30870 (1850,69270)	1210 (670,2420)	1680 (720,1880)
Ethylbenzene [pptv]	640 (250,950)	220 (40,530)	150 (80,280)	170 (120,290)
Styrene [pptv]	1130 (670,1950)	800 (290,1180)	280 (140,1110)	360 (200,820)
*n-*Propylbenzene [pptv]	40 (20,70)	40 (20,110)	20 (5,40)	30 (15,70)
Butanal [pptv]	169900 (60100,238800)	0 (0,8900)	700 (200,17100)	600 (0,34500)
Hexanaldehyde [pptv]	1250 (700,3800)	485 (275,1600)	450 (250,3050)	575 (175,2150)
Butanone [pptv]	478800 (374600,707000)	339600 (219900,555700)	5600 (2300,15900)	9500 (6400,31200)
Unk M^§^	10530 (5841,20041)	12854 (7971,44834)	1095 (263,2664)	1633 (359,3345)

Data were presented with median and range (minimum, maximum).

^§^unknown peaks were presented as area under the curve.

**Table 3:** Gases mainly from whole human blood

**Gas**	**Category 5 Gases mainly from whole human blood (significantly higher in whole blood only compared to broth only)**			
	**Broth**		**Blood**	
		***+ E. coli***		***+ E. coli***
*n*-Pentane [pptv]	50 (0,160)	10 (0,165)	1220 (835,1900)	1625 (815,2440)
Methanol [ppbv]	48 (34,132)	62 (41,110)	300 (210,346)	378 (226,704)
n-Heptane [pptv]	0 (0,315)	150 (30,165)	370 (260,915)	700 (210,4305)
2,3,4-Trimethylpentane [pptv]	35 (0,130)	55 (0,205)	125 (105,275)	145 (130,365)
*alpha*-Pinene [pptv]	5 (0,15)	5 (0,60)	40 (20,220)	60 (15,370)
Methyl chloride (CH_3_Cl) [pptv]	1045 (800,1390)	1140 (675,1815)	2190 (1215,2670)	1890 (1235,3025)
Dibromomethane (CH_2_Br_2_) [pptv]	1 (0,2)	1 (0,2)	47 (11,75)	54 (10,106)
*i*-Pentane [pptv]	225 (0,870)	210 (0,510)	1400 (545,2395)	1310 (0,4770)

Data were presented with median and range (minimum, maximum).

In category 1, there were six VOCs observed to be significantly increased from *E. coli*-inoculated whole blood when compared to VOCs from whole blood alone. These VOCs include dimethyl sulfide, carbon disulfide, ethanol, acetaldehyde, methyl butanoate and an unidentified gas S (see Table 1 and Figure 1 for examples of this category). Ethanol was also emitted from the *E. coli* cultured in the LB broth; however, the concentrations were relatively small when compared to the *E. coli*-inoculated whole blood (see Figure 1B).

In contrast, ten VOCs (see Table 1, category 2 and 3) were significantly elevated from *E. coli* in LB broth (Figures 2 and 3 show several examples). Isoprene, carbonyl sulfide (OCS), and unidentified gas peak R1 were significantly elevated in the headspace of *E. coli* in LB broth. These gases detected in the headspace of whole blood, and *E. coli*-inoculated whole blood. However, emitted isoprene, carbonyl sulfide and unidentified gas peak R1 levels from whole blood and *E. coli*-inoculated whole blood were not different (see Figure 2). Interestingly, seven out of these ten VOCs were substantially lower in the presence of human whole blood (see category 3 and Figure 3). These VOCs included dimethyl disulfide, dimethyl trisulfide, methylpropanoate, 1-propanol, methylcyclohexane, and unidentified gas peaks R2, and Q. The emitted levels from whole blood and *E. coli*-inoculated whole blood were not different in these seven gases.

Table 2 lists 16 VOCs that were observed to be significantly higher in LB broth when compared to the levels in whole blood (category 4). The emitted levels of 14 out of total 16 gases in this category (except n-Propylbenzene and unidentified gas peak M) were decreased in the presence of *E. coli*. Human whole blood emitted trace amount of these gases, and the levels of these gases were not altered in the presence of *E. coli*. Butanone levels in four different conditions (in the LB broth, in the *E. coli* in the LB broth, human whole blood, and *E. coli*-inoculated whole blood) are presented in Figure 4 as an example.

Table 3 lists eight VOCs that mainly originated from human whole blood (category 5). The emitted levels of these eight VOCs were substantially higher in the whole blood only when compared to the levels in LB broth. Interestingly, co-incubation of *E. coli* in human blood did not alter the concentrations of the gases.

Many elevated whole blood compounds were also detected in the headspace of the blood-collecting vacutainer and were rejected as carried-over contaminants. These include: styrene, 3-methylpentane, n-hexane, cyclohexane, methylcyclopentane, and 2-methyl-2-butene.

## Discussion

Specific VOCs released by *E. coli* in either LB broth or human blood were collected and compared to the levels emitted from LB broth or blood alone. Headspace gas samples were collected after incubation for 24 hr at 37°C from 10 mL samples inoculated with (or without)10^6^*E. coli*. This study demonstrates 1) that cultivated *E. coli* in LB broth produces distinct gas profiles, 2) for the first time the ability to modify *E. coli*-specific gas profiles by the addition of whole human blood (i.e., Methyl Propanoate was highly emitted from *E. coli* in LB broth, but barely detected in human whole blood), and 3) of most significance, that the data suggests that *E. coli*-human whole blood interaction has different gas emission profiles that can be used as non-invasive markers of *E. coli* infection (i.e., methyl butanoate).

The co-culture of *E. coli* in human whole blood emitted appreciable amounts of dimethyl sulfide when compared to the level from naive *E. coli* in the LB broth (Figure 1A). Dimethyl sulfide is a sulfur-containing odorous gas known to be released from marine phytoplankton and algae, and its release has been reported to protect algae and phytoplankton against environmental changes in salinity, radiation, and temperature [20,21]. Dimethyl sulfide released from damaged phytoplankton has also been proposed to act as a deterrent, or chemical weapon, against consumption by zooplankton [22]. In addition, it has been proposed that some microorganisms are able to generate gaseous dimethyl sulfide from dimethylsulphoniopropionate [23-25]. A significant concentration of dimethyl sulfide was observed in the headspace of *E. coli*-inoculated whole blood, whereas the headspace of *E. coli* in LB broth contained much less dimethyl sulfide (Figure 1A). This is the first observation that *E. coli*-inoculated human whole blood produces dimethyl sulfide. Allardyce and colleagues reported that dimethyl sulfide was detected from *E. coli* culture using BacT/ALERT, which contains a mixture of media with blood [8,26]. It is interesting to speculate the possible roles for dimethyl sulfide. Considering that dimethyl sulfide is a cryoprotectant for algae, dimethyl sulfide could be a mediator of survival of blood cells in response to the stresses of bacterial infection or the response of *E. coli* in the face of the bactericidal components of whole blood. Other studies utilizing different media/broth to culture *E. coli* did not observe dimethyl sulfide in the headspace above the culture [9,13].

Various strains of *E. coli* have been reported to metabolize glucose (and other sugars) to generate ethanol regardless of culture medium or broth used [3,27-34]. In the current study, ethanol has been detected in *E. coli* in LB broth and in the presence of human whole blood (Figure 1B). *E. coli* in LB broth emitted greater than 8 ppmv ethanol. The emission level was even greater (>15 ppmv) when *E. coli* was mixed with human blood. It is likely that the ethanol emission from *E. coli* depends on the available glucose in the LB broth or human whole blood.

Acetaldehyde has previously been detected in exhaled human breath [35,36] and in human lung cancer cell line cultures [37-39]. We previously demonstrated that both malignant myelocytic white blood cells such as HL60 and neutrophils are capable of producing acetaldehyde (16;18). The current study also demonstrates that *E. coli*-inoculated whole blood obtained from healthy subjects emitted appreciable amounts of acetaldehyde into the gas phase greater than the emission from whole blood (Table 1). In addition, acetaldehyde has been detected from other strains of *E. coli* and other microorganisms such as *Pseudomonas* cultured in human blood and medium mixture [8,26]. The LB broth itself also emits some acetaldehyde. However, the levels were not altered in the presence of *E. coli* in LB broth. This observation and others may suggest that acetaldehyde is likely a metabolic byproduct of the interaction between *E. coli* and the various components of whole blood [40,41].

Other VOCs generated only in the *E. coli-*inoculated whole blood were methyl butanoate and a gas that remains unidentified, unknown S. Methyl butanoate has been detected in the exhaled breath of patients with liver disorders [42]. These gases have the potential to be breath biomarkers of *E. coli* infection since these VOCs only appear in the *E. coli*-inoculated whole blood.

There were four VOCs found in the headspace above cultured *E. coli* in LB broth that were significantly lower in the presence of human whole blood. These VOCs include 1-propanol and the unknown VOCs R1, R2, and Q (see Table 1). Note that both LB broth and human whole blood generate negligible amounts of these VOCs. It remains unclear whether these VOCs are produced by *E. coli* and metabolized by certain components in the blood or the gas production itself was suppressed in the blood. One such possible mechanism for 1-propanal would be the production of 1-propanol by *E. coli*[43] and then, when co-cultured with whole blood, 1-propanol is metabolized by alcohol dehydrogenase in the human whole blood [44]. These compounds may prove interesting in cellular mechanistic studies as they have potentially been catabolised by *E. coli* and/or whole blood.

Isoprene, carbonyl sulfide, and unidentified gas peak R1 were higher in the headspace of *E. coli* in LB broth when compared to the LB broth only. carbonyl sulfide has been detected in the human breath [14]. Breath carbonyl sulfide concentrations were significantly higher in subjects with cystic fibrosis and it has been suggested that it originates from respiratory bacterial colonization. In effect, subjects with poorer pulmonary function tended to have greater breath carbonyl sulfide concentrations [14]. Isoprene has also been detected in exhaled human breath, and its possible association with blood cholesterol levels [45,46] and end-stage renal failure [47] has been suggested. In addition, various bacterial species, including *E. coli,* have been reported to produce isoprene, though emitted isoprene levels varied depending on the culture medium [48]. In the current study, *E. coli* contributes to isoprene emission with median value of 1600 pptv when cultured in the LB broth. Human whole blood obtained from healthy subjects emitted a median value of 1000 pptv of isoprene. Interestingly, *E. coli* inoculation into the whole blood does not affect emitted isoprene levels. This observation may suggest that isoprene release may be governed by different mechanisms when *E. coli* is cultured in the LB broth and human whole blood.

In contrast, there are certain VOCs that originated from LB broth only or human whole blood only that are not increased by the addition of *E. coli* (Table 2, category 4 and Figure 4; Table 3, category 5 and Figure 5). For example, butanone (an exemplary gas, category 4) and *i*-Pentane (an exemplary gas, category 5) emissions were observed either in LB broth only or human whole blood only, respectively. However, the emission levels were not altered in the presence of *E. coli*; the VOCs under these categories were not considered of interest in searching for *E. coli* -associated gas biomarkers.

## Conclusions

The current study demonstrates that cultivated *E. coli* produces distinct gas profiles depending on the medium used (i.e., LB broth or human whole blood). In addition, and of most significance, the data suggests that the interaction between *E. coli* and human whole blood presents a different gas emission profile that may have the potential to be used as non-invasive marker of *E. coli* infection. Additional investigations will be required to provide a more specific understanding of how the immune response of whole blood specifically alters the gas signature of invading *E. coli.* These data also provide the beginning of an inventory of VOCs associated with *E. coli* –the necessary first step from which an exploration of other pathogenic microorganisms can follow.

## Abbreviations

VOCs: Volatile organic compounds; E. coli: *Escherichia Coli*; DMS: Dimethyl sulfide; CS2: Carbon disulfide; OCS: Carbonyl sulfide; DMDS: Dimethyl disulfide; DMTS: Dimethyl trisulfide.

## Competing interests

The authors declare that they have no competing interests.

## Authors’ contributions

HWS and BJU designed and performed experiments and wrote the manuscript. SM participated in chemical analysis of volatile gases obtained from *E.coli* cultures. FPZ contributed experimental design and participated in culturing *E.coli*. SYL carried out statistical analysis. DMC and DRB participated in the design of the experiments and provided a review of the manuscript. All authors read and approved the final manuscript.
